# “Airway-First, Heart-Early” Strategy: Managing Neonatal Dextro-Transposition of the Great Arteries in Pierre Robin Sequence

**DOI:** 10.7759/cureus.88928

**Published:** 2025-07-28

**Authors:** Fatima Abeer, Ali Ahraz Wani, Aisha Mahmood Ul Hassan, Aasim Ayaz Wani, Gazala Andleeb

**Affiliations:** 1 Department of Medicine, Government Medical College, Srinagar, Srinagar, IND; 2 Department of Pediatrics, Government Medical College, Srinagar, Srinagar, IND; 3 Department of Biostatistics, National Institute of Technology, Srinagar, Srinagar, IND

**Keywords:** arterial switch operation (aso), lecompte maneuver, longitudinal case report, neonatal congenital heart disease, pediatric cardiac surgery outcomes, pierre robin sequence, postoperative prognosis, retrognathia, syndromic airway obstruction, transposition of the great arteries (tga)

## Abstract

The concurrent presentation of Pierre-Robin sequence (PRS) and dextro-transposition of the great arteries (d-TGA) is extremely rare and presents distinct management challenges. We describe a term male neonate with the classic PRS triad (severe micrognathia, glossoptosis, and a U-shaped cleft palate) who developed profound cyanosis unresponsive to supplemental oxygen. Echocardiography demonstrated d-TGA with an intact ventricular septum, a restrictive patent foramen ovale, and a patent ductus arteriosus. Prone positioning and a nasopharyngeal airway were unsuccessful in improving oxygenation. On day 7 of life, tongue-lip adhesion relieved glossoptosis and increased arterial oxygen saturation to >94 %. Definitive arterial switch repair was performed on day 10 while the infant received prostaglandin E₁. Post-operative care involved coordinated input from cardiology, nephrology, and infectious-disease teams to manage transient renal dysfunction and ventilator-associated pneumonia, both of which resolved. The infant was discharged hemodynamically stable on day 33 and, at 15-month follow-up, exhibited normal neurodevelopment, a left-ventricular ejection fraction of 60%, and growth at the 75th percentile. This case supports an “airway-first, heart-early” strategy: prompt, reversible airway stabilization followed by timely arterial switch operation can achieve outcomes comparable to isolated d-TGA, even in resource-limited settings, when coupled with structured multidisciplinary care and vigilant postoperative surveillance.

## Introduction

The simultaneous occurrence of Pierre Robin sequence (PRS) and dextro-transposition of the great arteries (d-TGA) constitutes a rare neonatal emergency that poses intricate management challenges [1]. PRS (incidence 1 per 8,500-14,000 live births) is defined by micrognathia, glossoptosis, and frequently a U-shaped cleft palate, all arising from disrupted mandibular development during gestational weeks 7-10 [2]. Recent work implicates genetic variants near SOX9, a master regulator of neural-crest-cell (NCC) migration critical to both craniofacial and cardiac morphogenesis; murine SOX9 knockouts develop defects reminiscent of d-TGA [3]. Other loci, such as BMPR1B and KCNJ2, have been linked to isolated or syndromic PRS but not to the combined PRS-d-TGA phenotype [3, 4]. d-TGA, with an incidence of 1 per 3,500 births, arises from abnormal rotation of the truncus arteriosus during weeks 5-6, producing ventriculo-arterial discordance and parallel systemic and pulmonary circuits. Without mixing through a patent ductus arteriosus (PDA) or patent foramen ovale (PFO), profound hypoxemia ensues [5]. Only a handful of PRS-d-TGA cases have been documented [1]; in these infants, airway obstruction and cyanosis coexist from birth, and glossoptosis further intensifies hypoxia [4,6].

Diagnosis demands high clinical suspicion. Clinicians must evaluate PRS stigmata (micrognathia, stridor) alongside cardinal d-TGA findings (cyanosis refractory to supplemental O₂); echocardiography can be technically challenging owing to mandibular hypoplasia. Management hinges on securing the airway before the arterial switch operation (ASO). Prostaglandin E₁ infusion and fiber-optic intubation are often required to maintain ductal patency and minimize procedural risk [7,8]. Emerging evidence for shared embryologic roots, including variants in SOX9 and KMT2D, suggests a unifying developmental mechanism behind the dual phenotype, underscoring the need for further investigation [6, 9].

## Case presentation

Initial presentation and assessment

**Figure 1:**
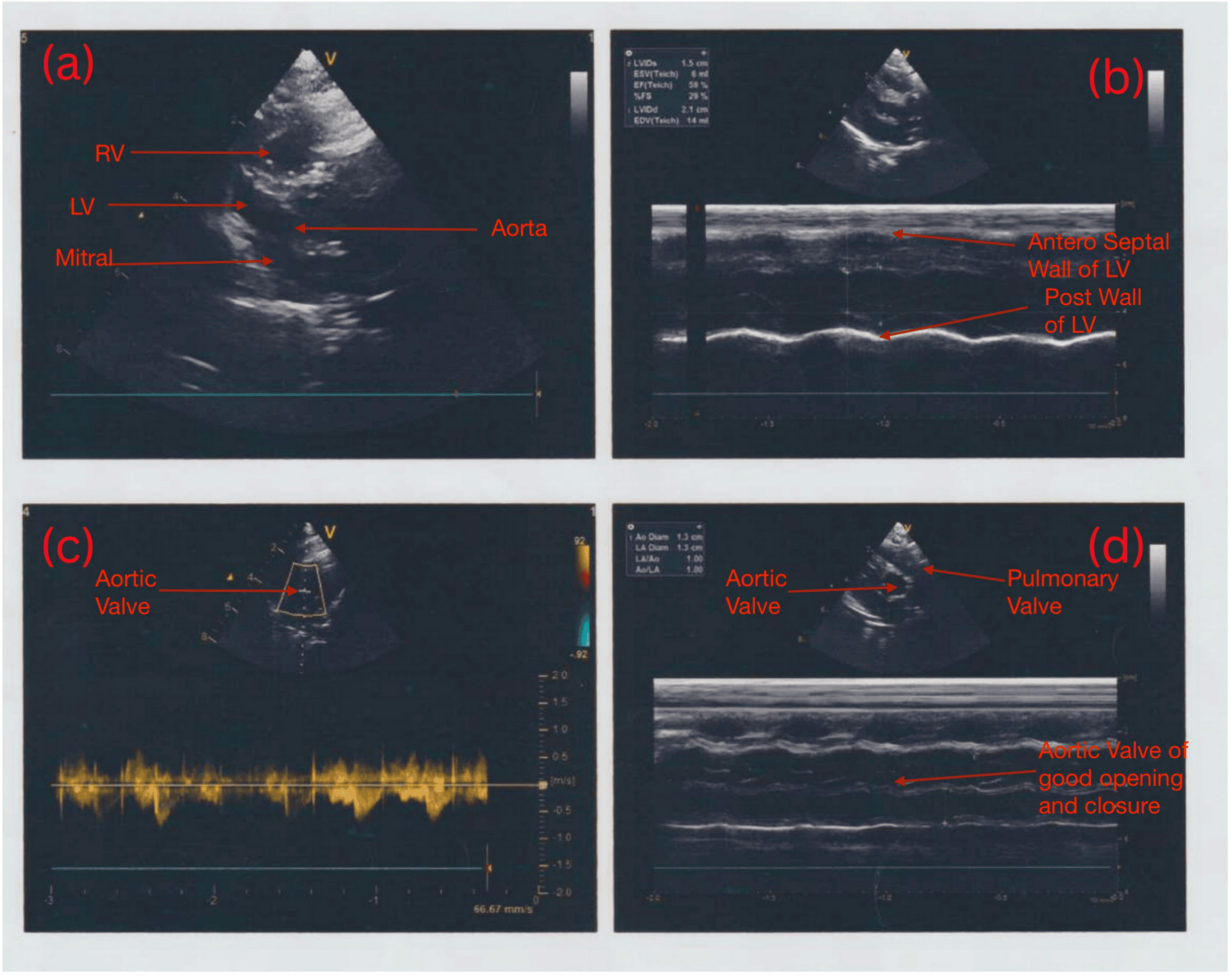
Post-operative transthoracic echocardiogram after arterial switch operation (a) Parasternal long-axis view demonstrating preserved left ventricular (LV) geometry, with visualization of the left and right ventricles (RV), aorta, and mitral valve. (b) M-mode tracing of the left ventricle, illustrating normal anterior septal and posterior wall motion. (c) Doppler echocardiogram demonstrating flow through the aortic valve. (d) M-mode tracing of the aortic valve and pulmonary valve, clearly showing normal aortic valve opening and closure, highlighting excellent post-repair function.

A 3.7 kg male neonate was delivered at 39 weeks’ gestation via spontaneous vaginal birth at a district hospital in Budgam, Jammu and Kashmir, India. The pregnancy had been uneventful with routine prenatal care, but no detailed fetal-anomaly scan was performed. Within minutes of birth, the infant developed profound central cyanosis: peripheral oxygen saturation remained 75-82% despite supplemental oxygen. Physical examination revealed the classic PRS triad--pronounced micrognathia with mandibular recession, glossoptosis with posterior tongue displacement causing visible upper-airway obstruction, and a U-shaped soft-palate cleft (Figure 1). He exhibited severe respiratory distress, paradoxical chest-wall motion, and intermittent apnoea during feeding attempts. Arterial blood gas on room air showed mixed respiratory and metabolic acidosis (pH 7.29, PaCO₂ 55 mm Hg, PaO₂ 47 mm Hg) with lactate 3.30 mmol/L, indicating inadequate tissue oxygenation. Conservative airway measures such as prone positioning, humidified oxygen, and nasogastric decompression provided minimal relief.

**Figure 2:**
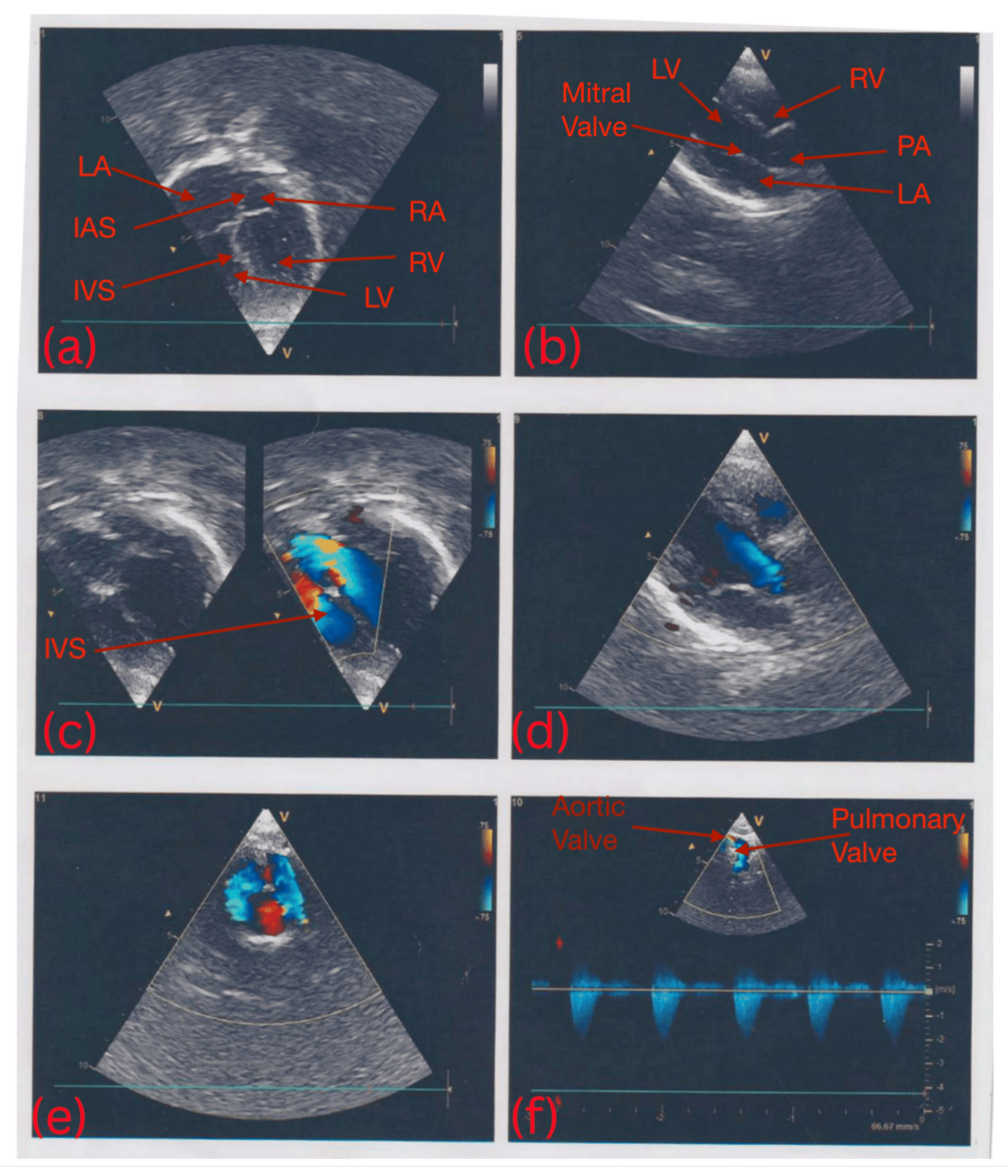
Echocardiographic views before surgical intervention. (a) A four-chamber view demonstrating the left atrium (LA), left ventricle (LV), right atrium (RA), and right ventricle (RV), with intact interatrial septum (IAS) and interventricular septum (IVS). (b) A view showing the left atrium (LA) and left ventricle (LV) separated by the mitral valve, with the pulmonary artery (PA) arising from the left ventricle. (c) and (e) illustrate restricted intercirculatory mixing, likely indicating normal or near-normal blood flow patterns within the heart. (f) A suprasternal view of the echocardiogram clearly visualizing the aortic valve and the pulmonary valve.

Because the central cyanosis seemed out of proportion to the airway obstruction, a cyanotic congenital heart lesion such as d-TGA was suspected. Transthoracic echocardiography confirmed d-TGA, demonstrating abnormal great-vessel orientation and turbulent flow (Figure 2). The four-chamber view (Figure 2a) showed ventriculo-arterial discordance with the aorta arising from the right ventricle, while the suprasternal view (Figure 2f) displayed the parallel great-vessel configuration pathognomonic for d-TGA, excluding other conotruncal anomalies (e.g., double-outlet right ventricle).

Laboratory studies revealed an elevated triiodothyronine level (T₃ 2.50 ng/mL, Table 1), suggesting a hypermetabolic state not typical of isolated d-TGA and likely reflecting the combined effects of chronic hypoxaemia and increased cardiac workload from parallel circulation. The complete blood count revealed microcytic anemia (Hgb 9.7 g/dL, MCV 75.6 fL) and thrombocytosis (438 × 10³/μL). The elevated eosinophil count (6.1%) likely reflected a stress-related immune response or early allergen exposure (e.g., to prophylactic antibiotics), as parasitic or atopic etiologies were excluded through clinical history and examination. The white cell count was normal, excluding sepsis or acute marrow suppression.

**Table 1:** Thyroid Function Test (Immunology Panel)

Parameter	Value	Unit	Reference Range
Tri‑iodothyronine (T₃)	2.5	ng/mL	0.80 – 2.10
Thyroxine (T₄)	11.9	µg/dL	5.10 – 14.06
Thyroid-stimulating hormone (TSH)	1.7	µIU/mL	0.27 – 5.20

**Table 2:** Complete Blood Count (CBC)

Parameter	Value	Unit	Reference Range
WBC	5.18	×10³/µL	5.0 – 19.5
Neutrophils	34.1	%	20 – 40
Lymphocytes	49.4	%	40 – 70
Monocytes	9.7	%	2 – 10
Eosinophils	6.1	%	1 – 4
Basophils	0.7	%	<1
Red Blood Count (RBC)	3.79	×10⁶/µL	4.1 – 5.5
Hemoglobin (HGB)	9.7	g/dL	10.5 – 14.0
Hematocrit (HCT)	28.6	%	32 – 44
Mean corpuscular volume (MCV)	75.6	fL	70 – 86
Mean corpuscular hemoglobin (MCH)	25.6	pg	23 – 31
Mean corpuscular hemoglobin concentration (MCHC)	33.9	g/dL	30 – 35
Red cell distribution width–CV (RDW‑CV)	19.6	%	<14.5
Platelet Count	438	×10³/µL	150 – 400
Mean platelet volume (MPV)	9.1	fL	6.5 – 10.5

## Discussion

The concurrent presentation of d-TGA and PRS represents an extraordinarily rare clinical entity with few cases reported in the medical literature. This unique combination creates complex diagnostic and therapeutic challenges that require a systematic, prioritized approach to achieve successful outcomes.

Embryological origins and genetic considerations

The rarity of this combined presentation underscores the distinct embryologic origins and developmental timelines of the two anomalies. D-TGA arises from conotruncal maldevelopment during gestational weeks 5-6, whereas PRS stems from mandibular hypoplasia that unfolds between weeks 7-10 [8]. This temporal separation implies that their coexistence is either coincidental or the result of upstream molecular perturbations that simultaneously influence neural-crest-cell migration and differentiation. Recent genomic studies lend weight to the latter hypothesis by identifying mechanistic links between craniofacial and cardiac morphogenesis [9]. Variants in SOX9 disrupt both mandibular formation and cardiac neural-crest-cell function, while mutations in KMT2D alter chromatin-remodeling processes essential for facial and cardiac organogenesis [10]. The clinical findings in our patient add further evidence to these proposed genetic connections and reinforce the importance of comprehensive genetic evaluation in similar cases.

"Airway-first, heart-early" management paradigm 

This case reinforces a crucial management tenet: in syndromic congenital heart disease, the airway must be secured before cardiac intervention. At birth, glossoptosis concealed the underlying cyanotic lesion, postponing echocardiographic confirmation until 48 hours of life and allowing progressive hypoxaemia and metabolic acidosis (lactate 3.3 mmol/L) to develop. Tongue-lip adhesion on day 7 proved decisive: by eliminating the upper-airway obstruction, it raised oxygen saturation to >94 % on moderate supplemental oxygen, established the physiologic stability required for safe surgery, and preserved the ideal timing for the arterial switch operation. Because the procedure is reversible, it avoids tracheostomy-related morbidity while maintaining the period of left-ventricular conditioning to systemic pressures. The arterial switch was performed on day 10 and then proceeded uneventfully, despite a 1LCx/2R coronary anatomy.

Complications in syndromic congenital heart disease

The postoperative course underscored how syndromic presentations can be markedly more complex than isolated cardiac lesions. Acute kidney injury developed early and necessitated peritoneal dialysis for six days, approximately twice the duration typically reported after stand-alone d-TGA repairs. This prolonged renal dysfunction likely reflected the additive impact of cardiopulmonary-bypass inflammation, the chronic hypoxaemic stress of parallel circulation, and exposure to nephrotoxic medications. Infectious morbidity also proved significant. On postoperative day 20, the infant developed ventilator-associated pneumonia caused by Acinetobacter baumannii, illustrating the heightened vulnerability of syndromic cardiac patients to nosocomial pathogens; comparable infections are documented in 5-15 % of complex congenital heart-disease cases. Nutritional recovery lagged behind cardiopulmonary stabilization. Because of persistent retrognathia and palatal incompetence, the patient did not achieve full oral feeding until postoperative day 27, a timeline consistent with reports that up to half of PRS infants struggle with feeding in the absence of specialized interventions. Microcytic anemia (Hb 9.7 g/dL) from PRS-related malnutrition reduced oxygen-carrying capacity, exacerbating tissue hypoxia. Iron supplementation was deferred until post-ASO due to infection risk.

Resource-limited setting considerations

This experience underscores that, even in resource-constrained settings, favorable outcomes can be secured when evidence-based protocols are applied systematically. Early detection is paramount: instituting universal transthoracic echocardiography within the first 24 hours of life for neonates who display major craniofacial anomalies permits timely recognition of otherwise occult cardiac lesions [11, 12]. Once an airway obstruction is confirmed, surgical strategy should favor reversible procedures such as tongue-lip adhesion rather than primary tracheostomy, thereby preserving the window for optimally timed cardiac repair. After surgery, rigorous surveillance protocols-encompassing standardized hemodynamic, renal, and infectious-risk monitoring-allow emerging organ dysfunction to be identified and addressed before it becomes irreversible. Critically, the coordinated involvement of cardiac surgery, nephrology, infectious disease, otolaryngology, and nutrition teams transformed potentially fatal complications into ordinary, manageable clinical challenges, demonstrating the power of multidisciplinary care when guided by structured, protocol-driven management.

Long-term outcomes and prognosis

At 15-month follow-up, the child exhibits an excellent recovery profile: left-ventricular ejection fraction has normalized to 60 %, and somatic growth tracks at the 75th percentile. Neurodevelopmental milestones are age-appropriate, indicating that neither the early period of profound hypoxemia nor exposure to cardiopulmonary bypass inflicted discernible neurological sequelae. These findings suggest that, when managed with timely airway stabilization, precise surgical intervention, and vigilant multidisciplinary care, the long-term prognosis for syndromic d-TGA can nearly match that of isolated cardiac malformations [12, 13].

Study limitations and future directions

This single-patient report carries two principal limitations: the absence of comprehensive genetic testing and a relatively short follow-up period. Advanced genomic sequencing was unavailable at our institution, a common constraint in resource-limited settings where urgent clinical management takes precedence and diagnostic breadth is curtailed. As a result, we cannot clarify whether shared molecular pathways underlie the co-occurrence of craniofacial and cardiac malformations in this infant. Neurodevelopmental surveillance, although reassuring at 15 months, began with formal Bayley-III testing only at that point. Earlier and serial administrations might reveal subtler cognitive or motor deficits, particularly in children who endured prolonged neonatal hypoxemia. Continued longitudinal monitoring of neurodevelopment and cardiac performance, therefore, remains essential.

Looking ahead, several research directions emerge. First, future cases should incorporate broad molecular sequencing to identify pathogenic variants and illuminate developmental pathways, especially those governing neural-crest cell migration and signaling, that could link PRS and d-TGA. Second, an international registry would facilitate standardized collection of anatomic variables, timing of interventions, and long-term outcomes across cardiovascular and neurodevelopmental domains, enabling meaningful comparisons and pooled analyses. Third, follow-up protocols after an arterial switch operation should include serial echocardiographic assessment of neo-aortic root dimensions indexed to body-surface area and evaluation of coronary-ostial patency to detect late complications. Finally, in regions where universal neonatal echocardiography is impractical, a targeted screening strategy that prioritizes infants with craniofacial anomalies, refractory hypoxemia, or feeding intolerance could maximize diagnostic efficiency without sacrificing sensitivity.

The clinical course described here illustrates that the rare conjunction of PRS and d-TGA can be managed successfully with an “airway-first, heart-early” paradigm. Neither anomaly was detected prenatally, underscoring the need for meticulous postnatal assessment when craniofacial abnormalities and central cyanosis coexist. Tongue-lip adhesion, chosen over mandibular distraction for its rapid and reversible airway stabilization, permitted a safe arterial switch repair on day 10 despite the presence of an intact ventricular septum. Profound cyanosis with initial oxygen saturations in the mid-50 percent range did not necessitate balloon atrial septostomy because the foramen ovale remained non-restrictive. Postoperative feeding progressed from nasogastric to oral intake under speech-language pathology guidance, and multidisciplinary collaboration among cardiology, craniofacial surgery, neonatology, anesthesiology, and nutrition specialists was indispensable throughout the perioperative period. At 15 months, the child demonstrates normal cardiac function, appropriate somatic growth, and age-appropriate neurodevelopment, affirming the effectiveness of this management approach even in syndromic presentations. Our experience has prompted the creation of a standardized institutional pathway mandating early multispecialty involvement for neonates with concurrent airway and cardiac anomalies. This model, if integrated into regional congenital-anomaly networks, could help ensure timely referral and optimized care regardless of geographic or economic barriers.

## Conclusions

The concurrent presentation of PRS and d-TGA represents a rare and life-threatening neonatal emergency requiring precise, time-sensitive intervention. This case underscores the success of a strategic, multidisciplinary "airway-first, heart-early" approach, where early reversible airway stabilization via tongue-lip adhesion, particularly when severe glossoptosis causes refractory hypoxia (SpO₂ <90% despite positioning) or delays cardiac surgery-enabled definitive arterial switch repair.

While challenges like mobile ICU availability persist, the core model (protocolized screening, reversible interventions, and multidisciplinary task-sharing) remains replicable. Ultimately, this report demonstrates that even rare, high-risk anomalies can be managed successfully in underserved communities through evidence-based, resource-conscious strategies. Investing in surgical infrastructure and regional referral systems, while acknowledging the limitations of bedside diagnostics, will expand access to life-saving care for syndromic neonates.
